# External Biliary Fistula

**DOI:** 10.1155/1998/42791

**Published:** 1998

**Authors:** J. Dadoukis, J. Prousalidis, D. Botsios, E. Tzartinoglou, S. Apostolidis, V. Papadopoulos, H. Aletras

**Affiliations:** 1 4th Surgical Clinic School of Medicine Aristotelian University of Thessaloniki Thessaloniki Greece; 2 1st Surgical Propedeutic Clinic School of Medicine Aristotelian University of Thessaloniki Thessaloniki Greece

## Abstract

We report 210 cases of external biliary fistula treated in our clinics between 1970–1992. In 7 cases, fistulas were formed after iatrogenic bile duct injury, in 4 cases after exploration of common bile duct, in 4 cases due to disruption of biliary-intestinal anastomosis, and in 2 cases due to liver trauma. In 85 cases bile leak was observed after cholecystomy, in 103 cases after hydatid disease surgery, and in 4 cases after the passage of P.T.C. catheter. In one patient the appearance of the fistula was due to spontaneous discharge of a gallbladder empyema. 173 cases were managed conservatively, and 37 cases surgically.


*HPB Surgery*, 1998, Vol. 10, pp. 375–377
Reprints available directly from the publisher
Photocopying permitted by license only

© 1998 OPA (Overseas Publishers Association)
Amsterdam B.V. Published under license
under the Harwood Academic Publishers imprint,
part of The Gordon and Breach Publishing Group.
Printed in India.

### External Biliary Fistula

J. DADOUKIS[a], J. PROUSALIDIS[b], D. BOTSIOS[a], E. TZARTINOGLOU[b], S. APOSTOLIDIS[b], V. PAPADOPOULOS[b] and H. ALETRAS[b]

[a]*4th Surgical Clinic;* [b]*1st Surgical Propedeutic Clinic, School of Medicine, Aristotelian University of Thessaloniki Thessaloniki, Greece*

*(Received 7 July 1996; In final form 28 January 1997)*

**We report 210 cases of external biliary fistula treated in our clinics between 1970–1992. In 7 cases, fistulas were formed after iatrogenic bile duct injury, in 4 cases after exploration of common bile duct, in 4 cases due to disruption of biliary-intestinal anastomosis, and in 2 cases due to liver trauma. In 85 cases bile leak was observed after cholecystomy, in 103 cases after hydatid disease surgery, and in 4 cases after the passage of P.T.C. catheter. In one patient the appearance of the fistula was due to spontaneous discharge of a gallbladder empyema. 173 cases were managed conservatively, and 37 cases surgically.**

*Keywords:* Billiary fistula

### INTRODUCTION

External biliary fistulas represent a well recognized complication of biliary surgery frequently necessitating laparotomy for its management [1, 2, 3]. By studying our series of external biliary fistulas we tried to provide some guidelines for the correct management of this difficult surgical problem.

### MATERIAL AND METHOD

Two hundred and ten patients with external biliary fistulas treated in the 1st Propedeutic and 4th Surgical Clinics from 1970 to 1992 were included in the study. Fistula formation occurred in 7 patients due to operative trauma of common bile duct, (5 during cholecystectomy, and 2 during gastrectomy), in 4 patients, after exploration of the common bile duct, (2 due to retained bile duct stone and 2 after removal of the T-tube), in 4 following disruption of biliary–intestinal anastomosis, in 2 after liver trauma. There were also 85 postcholecystostomy biliary fistula, 103 associated with hydatid syrgery, 4 due to passage of PTC catheter for malignant jaundice and one spontaneous fistula associated with suppurative cholecystitis. Tube cholangiography or fistulography, were performed in 180 of the patients, transhepatic percutaneous cholangiography or endoscopic procedures were useful in 6 and 10 cases relatively, and sonogram or computerized tomogram were done if the fistula was related to hydatid disease of the liver.

Correspondence to: J. PROUSALIDIS 1st Surgical Propedeutic Clinic Aristotelian University of Thessaloniki, A.H.E.P.A. Hospital, Thessaloniki, Greece.

### RESULTS

One hundred and seventy three patients were conservatively and 37 surgically managed. (Tabs. I, II).

In the cholecystomy group there were three postoperative deaths. One patient with emphysematous cholecystitis died on the third postoperative day from sepsis, another one with calculus cholecystitis on the tenth day from cardiorespiratory failure and the third, 90 years of age, morbidly obese with cholelithiasis, after 3 months of complicated hospitalization.

In the hydatid group three patients died one during his stay in the hospital and 2 after a period of time. One 60 year old obese patient, with multiple hydatid cysts of the liver, complicated with suppuration, died on the 20th postoperative day from sepsis. One patient, initially treated elsewhere for hydatid of the liver succumbed in our clinic 3 years later with secondary biliary cirrhosis. The patient who underwent the fistulojejunal anastomosis for biliary fistula, died 5 years later with bouts of cholangitis and secondary biliary cirrhosis.

The temporary deliberate external fistula in 3 of the 4 patients with cancer of proximal bile duct and jaundice remained in place, until their death after an average 6 months period.

Of the 185 survivors, follow-up period, ranged 2 to 24 years.

### DISCUSSION

External biliary fistula continues to be a problem. The persistent discharge of bile posto-

TABLE I Conservative management of external biliary fistulas

| | Patients | Time to closure |
|---|---|---|
| Iatrogenic | 3 (out of 7) | 10-30 days (mean 17) |
| * Postcholedochotomy | 3 (out of 4) | 3-45 days (mean 16) |
| Anastomotic leakage | 4 (out of 4) | 10-20 days (mean 15) |
| * Liver trauma | 2 (out of 2) | 15-20 days (mean 17) |
| Postcholecystostomy | 62 (out of 85) | 15-20 days (mean 18) |
| ** Hydatid surgery | 95 (out of 103) | 10 days-8 months |
| Interventional radiology | 4 (out of 4) | – |
| Total | 173 (out of 210) | |

* Spincterotomy (2 cases). Reinsertion of T-tube (1 case)
** Octreotide (2 cases)
** Octreotide (1 case).

TABLE II Operative management of external biliary fistulas

| | Patients | Time to operation |
|---|---|---|
| * Iatrogenic | 4 (out of 7) | 15-45 days (mean 20) |
| ** Postcholedochotomy | 1 (out of 4) | 3 days |
| *** Postcholecystostomy | 23 (out of 85) | 1-4 months (mean 3) |
| **** Hydatid disease surgery | 8 (out of 103) | 1, 5-6 months (mean 3) |
| ***** Spontaneous fistula | 1 (out of 1) | 2-3 days |
| Total | 37(out of 210) | |

* Hepatojejunal anastomosis
** Drainage of bile ascites
*** Cholecystectomy
**** Fistulojejunal anastomosis (1 case), distruption of omentoplasty (1case), replacement of the tube drainage (6 cases)
***** Cholocystectomy.

peratively is probably meaning an unrecognized surgical error or abnormal pathology around the biliary tree and results in unexpected prolongation of hospitalization [1, 2, 3]. Conservative management, as in this series, solve the problem in many cases otherwise an operation becomes mandatory [1, 4, 6, 7].

The treatment of "iatrogenic fistula" must be, undertaken by experienced surgeons. The delayed repair, as in our cases, with an biliodigestive anastomosis is generally easier than an early one. This is due to resolution of the local inflammation and dilatation proximal, to the stricture [2, 4, 5].

In the postcholedochotomy fistula endoscopic sphincterotomy as in our 2 cases, or endoscopic insertion of nasobiliary tube or endoprosthesis, are effective methods [1, 2, 6].

The management of postanastomotic leakage is usually easy, without operative intervention. The synthetic somatostatin analogue, inhibits bile secretion [1]. In two of our patients, it was useful.

In most the patients with a cholecystostomy and an unobstructed bile system, the fistula gradually resolves [3].

The hydatid surgery fistulas, in the absence of outflow obstruction in the distal bile duct, as in most of our cases, spontaneously stop [7]. We have noticed the beneficial role of octreotide in one and sphincterotomy in another of our patients. Total cystopericystectomy, not often feasible, or more radical operations obviate the complication of bile leakage. The mortality rate in this group was low and the time of death in the 3 patients was remote.

Biliary fistulation after intervation radiology for management of obstructive jaundice is considered a preventable ⟨⟨iatrogenic⟩⟩ complication [1]. As in our series, successful external transhepatic bile drainage remains permanent, or can be followed by a successful surgical or percutaneous endoprosthesis.

The single spontaneous external biliary fistula in our series, represents a rare situation [8]. Tratement is cholecystectomy with excision of the fistulous tract.

### *References*

[1] Czerniak, A. and Blumgart, L. H. (1987). External biliary fistula. *Surgery of the liver and biliary tract*, Vol. **1**, Blumgart L. H. pp. 895–907.

[2] Rosato, F. E. in Davis-Cristofer (1986). Gallstone ileus and fistula (1986). Textbook of surgery. The bilogical basis of modern surgical practice Sabiston. pp. 1161–1165.

[3] Winkler, E., Kaplan, O., Gutman, M., Skornick, Y. and Rozin, R. R. (1989). Role of cholecystostomy in the management of cirtically ill patients suffering from acute cholecystitis. *Br. J. Surg.*, **76**, 693–695.

[4] Zerniac, A., Thomson, J. N., Soreide, O., Benjamin, I. S. and Blumgart, L. H. (1988). The management of fistulas of the biliary tract after injury to the bile duct during cholecystectomy. *Surg. Gynecol. Obstet*, **167**, 33–38.

[5] Asbum, H. J., Ross, R. L., Lowell, J. A. and Munson, J. L. (1993). Bile duct injury during laparoscopic cholecystectomy : Mechanism of injury, prevention and management *World. J. Surg.*, **17**, 547–552.

[6] Feretis, C., Kekis, B., Bliouras, N., Shaheey, N. G., Daron, D. and Golematis, B. (1990). Postoperative external and internal biliary fistulas, unassociated with distal bile duct obstruction. Endoscopic treatment. *Endoscopy*, **22**, 211–213.

[7] Del Olmo, L., Merono, E., Moreira, V. F., Garcia, T. and Garcia-Plaza, A. (1988). Succesful tratment of postoperative external biliary fistuals by endoscopic spincterotomy. *Gastrointestinal endoscopy*, **34**, 307–309.

[8] Reed, M. W. and Tweedie, J. H. (1985). Spontaneous simultaneous internal and external biliary fistulas. *Br. J. Surg.*, **72**, 538.

